# Dipyrone and acetaminophen: correct dosing by parents?

**DOI:** 10.1590/S1516-31802007000100011

**Published:** 2007-01-04

**Authors:** João Guilherme Bezerra Alves, Fortunato José Cardoso, Camila Dornelas Câmara Almeida, Natalia Dornelas Câmara Almeida

**Keywords:** Fever, Antipyretics, Dipyrone, Acetaminophen, Dose, Cross-sectional studies, Febre, Antipiréticos, Dipirona, Acetaminofen, Dose, Estudos transversais

## Abstract

**CONTEXT AND OBJECTIVE:**

Several studies in developed countries have documented that a significant percentage of children are given inappropriate doses of acetaminophen and ibuprofen. The objective of this paper was to investigate parents’ accuracy in giving dipyrone and acetaminophen to their children, in a poor region.

**DESIGN AND SETTING:**

Cross-sectional study at the pediatric emergency department of Instituto Materno-Infantil Prof. Fernando Figueira, a teaching hospital in Pernambuco.

**METHODS:**

The inclusion criteria were age between 3 and 36 months, main complaint of fever and at least one dose of dipyrone or acetaminophen given to the child during the 24 hours preceding their arrival at the emergency department. The mothers were asked for demographic information and about the antipyretic doses given, which were compared with the recommended dosage.

**RESULTS:**

Among the 200 patients studied, 117 received dipyrone and 83 received acetaminophen. Overall, 75 % received an incorrect dose of antipyretic. Of the patients who received dipyrone, 105 (89.7%) were given an incorrect dose; 16 (15.2%) received too little dipyrone, and 89 (84.8%) received too much. Of the patients who received acetaminophen, 45 (54.2%) were given an incorrect dose; 38 (84.4%) received too little acetaminophen, and 7 (15.6%) received too much. There were no differences in maternal and child characteristics between the groups receiving correct and incorrect doses of medication, except for the type of medication (dipyrone versus acetaminophen).

**CONCLUSIONS:**

Most of the children treated were given inappropriate doses, mainly dipyrone overdosing and acetaminophen underdosing.

## RESUMO

**CONTEXTO E OBJETIVOS:**

Alguns estudos em países desenvolvidos têm demonstrado um elevado percentual de crianças que recebem doses inapropriadas de acetaminofen e ibuprofeno. O objetivo deste estudo foi verificar, em uma área carente, a acurácia dos pais na administração da dipirona e do acetaminofen a seus filhos.

**TIPO DE ESTUDO E LOCAL:**

Foi realizado um estudo transversal no departamento de Emergência do Instituto Materno-Infantil Professor Fernando Figueira, em Pernambuco.

**MÉTODOS:**

Foram admitidas à pesquisa crianças com idade entre 3 a 36 meses, com queixa principal de febre e o uso mínimo de uma dose de dipirona ou acetaminofen nas 24 horas que precederam sua chegada na emergência. Foi aplicado um questionário às mães sobre dados demográficos e o uso de antipiréticos. A dose administrada foi comparada com a recomendada.

**RESULTADOS:**

Dos 200 pacientes estudados, 117 receberam dipirona e 83 acetaminofen. 75% deles receberam doses incorretas. Dos pacientes que receberam dipirona, 105 (89,7%) usaram uma dose incorreta; 16 (15,2%) subdosagens e (84,8%) superdosagens. Dos pacientes que receberam acetaminofen, 45 (54,2%) utilizaram uma dose incorreta, 38 (84,4%) sub-dosagens e 7 (15,6%), super-dosagens. Não houve diferenças nas características maternas e das crianças que receberam doses corretas ou incorretas dos antipiréticos, exceto para o tipo de medicação, mais freqüentemente a dipirona.

**CONCLUSÕES:**

A maioria das crianças recebeu doses inapropriadas de antipiréticos, principalmente superdosagens de dipirona e subdosagens de acetaminofen.

## INTRODUCTION

Fever is a common reason for pediatric emergency department visits. Although in a large number of cases it is a benign and self-limiting condition, parents are often concerned about the perceived risks of convulsions and severe diseases.^1^ Parents’ misconceptions about fever as a disease with potentially devastating consequences lead to unnecessary use of antipyretics, especially during the first year of life.^2^ The Avon Longitudinal Study of Pregnancy and Childhood, performed in the United Kingdom, found that 84% of babies have been given some kind of antipyretic by the end of their first year of life.^3^

Several studies in developed countries have documented that a significant percentage of children are given inappropriate doses of antipyretics, which predictably result in incomplete lowering of temperature. This also leads to unnecessary use of healthcare providers and is an important issue for health professionals and policymakers.^4,5^

We did not find any articles in Medline or Lilacs (Literatura Latino-Americana e do Caribe em Ciências da Saúde) on home dosing of antipyretics among children who live in developing countries. Our hypothesis was that poor regions with populations of low sociocultural and economic level, and especially with high illiteracy rates, could favor widespread use of inappropriate doses of antipyretics.

## OBJECTIVE

The aim of this study was to investigate parents’ accuracy in giving the antipyretic drugs dipyrone and acetaminophen to their children, in a poor region.

## METHODS

A cross-sectional study was conducted among patients presenting to the pediatric emergency department at Instituto Materno Infantil Professor Fernando Figueira (IMIP), a teaching hospital in the State of Pernambuco, in northeastern Brazil, with an annual pediatric census of 30,000. Enrollment took place during a six-month period from September 2004 to February 2005. Patients were enrolled as a convenience sample.

The primary endpoint of the study was to determine the prevalence of incorrect dosing of dipyrone and acetaminophen. Approximately 200 patients were need to detect an expected misdosing prevalence based on a 15% incidence,^6^ taking an estimation error of 5% and a confidence level of 95%. To calculate the sample size, the following formula was used:


$$\text{n}={\left(\text{z/e}\right)}^{2}\text{p}\left(1-\text{p}\right)$$

The parameters used in this formula were: z = 1.96; e = 0.05; and p = 15%.

The inclusion criteria were maternal schooling of less than four years; per capita income of less than 1.00 United States dollar per day; age between three and 36 months; attendance on Monday to Friday from 5:00 p.m. to 9:00 p.m.; main complaint of fever; and at least one dose of dipyrone or acetaminophen given to the child during the 24 hours preceding arrival at the pediatric emergency department. Patients were excluded if their primary caregiver did not accompany them and/or refused consent, or did not complete the interview. For patients with multiple visits, only the first visit was included.

Questionnaires were applied to gather information that included the mother's age, number of years of schooling, per capita income, fever fear, fever control, the number of children in the household and the clinical examination. Parents were then asked about the last dose of antipyretics given prior to the hospital visit, including the drug type (dipyrone or acetaminophen), type of oral presentation (infant drops or elixir), dosing and side effects. The antipyretic dosage for that child was then compared with the recommended dosage: acetaminophen 10-15 mg/kg per dose and dipyrone 15-20 mg/kg per dose. The oral preparation types available in Brazil for dipyrone are infant drops 500 mg/ml and elixir 200 mg/5 ml; and for acetaminophen are infant drops 200 mg/ml and elixir 160 mg/5 ml.

Data were analyzed using Epi Info 6.0. The chi-squared test was used for comparison of categorical variables and Student's t test for comparison of continuous variables; p < 0.05 was considered significant. The study was granted approval by the Research Ethics Board of IMIP, and all the mothers gave their informed consent.

## RESULTS

No parent refused to be interviewed. The mothers’ and children's characteristics are shown in Table 1. Among the 200 patients studied, 83 received acetaminophen and 117 received dipyrone. Overall, 75% received an incorrect dose of antipyretic medication. Of the patients who received dipyrone, 105 (89.7%) were given an incorrect dose; 16 (15.2%) received too little dipyrone, and 89 (84.8%) received too much. Of the patients who received acetaminophen, 45 (54.2%) were given an incorrect dose; 38 (84.4%) received too little acetaminophen, and 7 (15.6%) received too much. No side effects were described by the mothers.

**Table 1.** Characteristics (n = 200) of mothers and children with the main complaint of fever

Variable	Mean (SD)	Number (%)
Mother's age (years)	25.4 (6.2)	-
Fever fear		200 (100.0)
Child's age (months)	15.3 (8.5)	-
Child's nutritional status		
Underweight		42 (21.0)
Normal		150 (75.0)
Overweight		8 (4.0)
Child's sex		
Male		114 (57.0)
Female		86 (43.0)

SD = standard deviation.

Analysis of the reports from parents who gave an incorrect dose (underdose or overdose) of dipyrone or acetaminophen, in comparison with those giving the recommended dose, showed that there were no significant differences with regard to fever control, medicine presentation (infant drops or elixir), the child's age and sex, and the mother's age. The only significant parameter was dipyrone use (Table 2).

**Table 2.** Comparison between number/percentage of parents who gave an incorrect dose and those who gave the recommended dose of dipyrone and acetaminophen to control fever in their children

		Correct dose (n = 50)	Incorrect dose (n = 150)	95% CI
		n (%)	n (%)	
Antipyretic	Dipyrone	12 (10.3)	105 (89.7*)	83.0 - 95.0
	Acetaminophen	38 (45.8)	45 (54.2*)	43.0 - 65.2
Fever control	Yes	35 (23.0)	117 (77.0)	69.5 - 83.4
	No	15 (31.2)	33 (68.8)	53.7 - 81.3
Presentation	Infant drops	45 (25.2)	133 (74.8)	67.7 - 80.9
	Elixir	5 (22.7)	17 (77.3)	54.6 - 92.2
Child's age	< 12 months	24 (27.2)	64 (72.3)	62.2 - 81.7
	≥12 months	26 (23.2)	86 (76.8)	68.0 - 84.2
Sex	Male	28 (24.5)	86 (75.5)	66.5 - 83.0
	Female	22 (25.5)	64 (74.5)	63.9 - 83.2
Mother's age	< 20 years	13 (27.6)	34 (72.4)	57.4 - 84.4
	≥20 years	37 (24.2)	116 (75.8)	68.2 - 82.4

CI = confidence interval;

* Chi-squared = 32.68; p < 0.001.

## DISCUSSION

Our study confirms the perception that parents frequently give the wrong dose of dipyrone and acetaminophen. More than half of the mothers gave an incorrect dose of antipyretic. In developed countries, Gribetz and Cronley^5^ found that 68% of patients underdosed acetaminophen, a proportion that was similar to what was found by Goldman and Scolnik and Li et al.^4,6^ Many factors may contribute to this inappropriate dosing. Parents may misunderstand the indications for and effects of antipyretics; there may be a lack of understanding of the different antipyretic preparations available; inaccurate measuring devices may be used; and parents may be u nable to determine and measure an appropriate dose.^7,8^ Moreover, parents frequently ascribe additional properties to antipyretic medications, falsely believing they provide antiviral and decongestant effects.^9^

“Fever phobia” and misconceptions about the harm caused by fever over 39° C are probably the main reasons why parents take their children to the emergency department.^1^ In our study, all the mothers said that they were afraid of fever, based on the belief that fever could cause convulsions.

Although other authors^4,8^ have associated improper dosage with patient age, patient weight and parental education, we were u nable to determine any patient demographic variables (age, sex or nutritional status) or caregiver demographic variables (age or fever fear) that would predict inappro priate home dosing. Use of dipyrone was the only variable that showed a significant difference between the two groups. Because of the risk of agranulocytosis, dipyrone (metamizole or noramidopyrine) has been banned in the United States, Canada, Japan, and many European countries, although the drug is available in many other parts of the world, including the Far East, Africa and Latin America.^10^ This antipyretic is widely used in Brazil and other countries in Latin America and its effectiveness and safety have recently been approved in Brazil and Mexico.^11,12^ One explanation for supratherapeutic dipyrone doses relates to the different preparations available. Dipyrone infant drops consist of 500 mg/ml, while acetaminophen drops consist of 200 mg/m. In Brazil, there is a very common popular habit of administering one drop of antipyretic per kilogram of weight. Children who receive supratherapeutic doses of dipyrone are at risk of hypothermia. This is why parents frequently say that “dipyrone reduces their children's blood pressure”.

No significant differences in fever control were found when analyzing reports from parents who gave incorrect doses of dipyrone or acetaminophen in comparison with those giving the recommended dose. We believe that the fact that most of the children who were improperly dosed had received too much dipyrone (84.8%) could explain these results. Moreover, these results were based only on parents’ reports and, in our environment, most of these people do not have a thermometer in their homes.^13^

There are some limitations to the pre sent study. First, it was limited to children presenting at the emergency department. Therefore, we have no data regarding subjects who received antipyretics at home and who did not seek a hospital evaluation. Intuitively, children treated with proper doses who defervesce would be less likely to present to the emergency department. Secondly, our sample size was small and geographically limited, and thus may not be representative of other patient populations.

## CONCLUSIONS

Despite the limitations of our study, several conclusions can be drawn. More than half of the children treated were given inappropriate doses, especially with overdo sing of dipyrone. Appropriate dosing was not associated with any patient or caregiver demographic variables. All the parents had concerns about fever. Emergency physicians should ask care-givers to carefully ascertain whether correct doses of antipyretics are being used at home. Additional research is needed to determine which factors interfere with improper use of antipyretics in develo ping countries.
